# Blood pressure and 10-year all-cause mortality: Findings from the PERU MIGRANT Study

**DOI:** 10.12688/f1000research.73900.1

**Published:** 2021-11-09

**Authors:** Aida Hidalgo-Benites, Valeria Senosain-Leon, Rodrigo M. Carrillo-Larco, Andrea Ruiz-Alejos, Robert H. Gilman, Liam Smeeth, J. Jaime Miranda, Antonio Bernabé-Ortiz

**Affiliations:** 1 Universidad Peruana de Ciencias Aplicadas, Lima, Peru; 2 Department of Epidemiology and Biostatistics, School of Public Health, Imperial College London, London, UK; 3 CRONICAS Center of Excellence in Chronic Diseases, Universidad Peruana Cayetano Heredia, Lima, Peru; 4 Department of International Health, Johns Hopkins Bloomberg School of Public Health, Johns Hopkins University, Baltimore, Maryland, USA; 5 Faculty of Epidemiology and Population Health, London School of Hygiene and Tropical Medicine, London, UK; 6 Department of Medicine, School of Medicine, Universidad Peruana Cayetano Heredia, Lima, Peru; 7 Universidad Científica del Sur, Lima, Peru

**Keywords:** Hypertension, pre-hypertension, blood pressure, mortality

## Abstract

Background

The long-term impact of elevated blood pressure on mortality outcomes has been recently revisited due to proposed changes in cut-offs for hypertension. This study aimed at assessing the association between high blood pressure levels and 10-year mortality using the Seventh Report of the Joint National Committee on Prevention, Detection, Evaluation, and Treatment of High Blood Pressure (JNC-7) and the American College of Cardiology and the American Heart Association (ACC/AHA) 2017 blood pressure guidelines.

Methods

Data analysis of the PERU MIGRANT Study, a prospective ongoing cohort, was used. The outcome of interest was 10-year all-cause mortality, and exposures were blood pressure categories according to the JNC-7 and ACC/AHA 2017 guidelines. Log-rank test, Kaplan-Meier and Cox regression models were used to assess the associations of interest controlling for confounders. Hazard ratios (HR) and 95% confidence intervals (95% CI) were estimated.

Results

A total of 976 records, mean age of 60.4 (SD: 11.4), 513 (52.6%) women, were analyzed. Hypertension prevalence at baseline almost doubled from 16.0% (95% CI 13.7%–18.4%) to 31.3% (95% CI 28.4%–34.3%), using the JNC-7 and ACC/AHA 2017 definitions, respectively. Sixty-three (6.4%) participants died during the 10-year follow-up, equating to a mortality rate of 3.6 (95% CI 2.4–4.7) per 1000 person-years. Using JNC-7, and compared to those with normal blood pressure, those with pre-hypertension and hypertension had 2.1-fold and 5.1-fold increased risk of death, respectively. Similar mortality effect sizes were estimated using ACC/AHA 2017 for stage-1 and stage-2 hypertension.

Conclusions

Blood pressure levels under two different definitions increased the risk of 10-year all-cause mortality. Hypertension prevalence doubled using ACC/AHA 2017 compared to JNC-7. The choice of blood pressure cut-offs to classify hypertension categories need to be balanced against the patients benefit and the capacities of the health system to adequately handle a large proportion of new patients.

## Introduction

Ischemic heart disease and cerebrovascular disease are the first and second cause of death globally. ^1^^,^^2^ Hypertension, as a cardiovascular risk factor, was the cause of 9.4 million deaths and is closely related to ischemic heart and cerebrovascular disease. ^3^ Worldwide, the number of adults living with hypertension has increased from 563 million in 1975 to 1.13 billion in 2015, and the prevalence of hypertension was estimated to be 24.1% and 20.1% in men and women, respectively. ^4^

Levels of blood pressure before the development of hypertension are known as pre-hypertension according to the Seventh Report of the Joint National Committee on Prevention, Detection, Evaluation, and Treatment of High Blood Pressure (known as JNC-7), ^5^ and those with pre-hypertension are more likely to develop hypertension and its consequences. In 2017, the American College of Cardiology and the American Heart Association (ACC/AHA 2017) changed the proposed cut-off points used for defining hypertension, and for instance, included part of the pre-hypertension cases as hypertension (known as stage I hypertension). ^6^ The adoption of the ACC/AHA 2017 guidelines may produce changes in the proportion of cases with hypertension as reported for the US general population by the SPRINT (Systolic Blood Pressure Intervention Trial) Study, where the prevalence of hypertension almost doubled from 49.7% using JNC-7 to 80.1% by ACC/AHA 2017. ^7^ Similar changes in hypertension prevalence have been described in different countries. ^8^^–^^13^

Different reports associate mean arterial and blood pressure levels with all-cause mortality and cardiovascular mortality. ^14^^–^^16^ Whilst the association between blood pressure levels, defined by JNC-7, and mortality has been well described, ^17^ the evidence of the impact of the new definitions of hypertension on all-cause mortality in resource-constrained settings remains limited. ^15^^,^^18^ Therefore, long-term studies involving populations from low- and middle-income countries (LMICs) are needed given that raised blood pressure is a major contributor to the global burden of disease. ^19^

This study aimed at assessing whether the levels of blood pressure, using two different guidelines, JNC-7 and ACC/AHA 2017, are associated with 10-year mortality using an ongoing Peruvian cohort study.

## Methods

### Study design

Data analysis of the PERU MIGRANT Study, a prospective ongoing cohort conducted enrolling three different population groups: rural, rural-to-urban migrants, and urban dwellers was carried out. ^20^ The baseline of the study was conducted in 2007–2008 and follow-ups were carried out in 2012–2013, 2015–2016, and 2018. ^21^ For this analysis, data from the baseline assessment and 2018 follow-up were used.

### Settings and participants

In Lima, a highly urbanized city, Las Pampas de San Juan de Miraflores was selected as the urban environment, whereas San Jose de Secce, a district of Ayacucho in the highlands, was selected as the rural site. Individuals, for any of the population groups, who were ≥ 30 years of age and habitual residents in the selected study sites were invited to participate at baseline. Rural dwellers were enrolled in San Jose de Secce, while urban residents and rural-to-urban migrants were recruited from Las Pampas de San Juan de Miraflores in Lima. ^20^ Pregnant women or potential participants unable to understand procedures and consent were excluded.

Participants were randomly selected using an age and stratified (30–39, 40–49, 50–59, and 60+) sampling approach, utilizing the most up-to-date census in the study area. San Jose de Secce (Ayacucho) was the area chosen for the selection of rural dwellers. Migrants were those born in Ayacucho but living in Las Pampas de San Juan de Miraflores (Lima) at the time of the study enrolment. Finally, urban dwellers were those permanently living in San Juan de Miraflores. ^20^

Power estimations were based on major risk factors in Huaraz (highlands) and Lima. The baseline study aimed at recruiting 1000 participants (200 in rural and urban groups, and 600 in the migrant group). Comparing Lima and highlands groups, the study had 84% power to detect a difference in the prevalence of hypertension (33% vs. 19.5%) enrolling 200 subjects in each group. Such power was 81% in the case of type 2 diabetes (7.6% versus 1.3%). ^20^

### Definition of variables

**Outcome** The outcome of interest was the time until an event, defined as the time, in years, lapsed from the baseline assessment (2007–2008) to death or censorship during follow-up. Information about vital status and date of death (or censoring) was obtained via assessment of the National Record of Identification and Civil Status (RENIEC (Spanish acronym)) conducted in 2018.

**Exposure** The exposure variable was hypertension-related categories using measurements of systolic blood pressure (SBP) and diastolic blood pressure (DBP) under two different definitions, JNC-7 and ACC/AHA 2017. Under the JNC-7 definition, ^5^ individuals were split into three categories: normal (SBP < 120 mm Hg and DBP <80 mm Hg without using specific medication), pre-hypertension (SBP 120–139 mm Hg and DBP 80–89 mm Hg without anti-hypertensive therapy), and hypertension (SBP ≥ 140 mm Hg or DBP ≥ 90 mm Hg, or those reporting previous diagnosis done by a physician or current anti-hypertensive treatment). On the other hand, under the ACC/AHA 2017 definition, ^6^ participants were split into four categories: normal (same as those in JNC-7), elevated blood pressure (SBP 120–129 mm Hg and DBP < 80 mm Hg, without medication), stage 1 hypertension (SBP 130–139 mm Hg and DBP 80–89 mm Hg without treatment), and stage 2 hypertension (same as those with hypertension in the JNC-7).

**Covariates** Other variables included as potential confounders in the analysis were: age (< 50 vs. ≥ 50 years), sex (men vs. women), education level (less than seven vs. more than seven years), socioeconomic status, defined by using an assets index and then split in tertiles (low, middle, high), and population group (rural, rural-to-urban migrant, and urban). In addition, behavioural variables were also included: daily smoking, self-reported, based on the consumption of at least one cigarette per day; alcohol use, defined according to the self-reported consumption of six or more beers (or equivalent) on the same occasion at least once a month (low vs. high); and physical activity level, based on the short version of the International Physical Activity Questionnaire (IPAQ) and split into low and moderate/high ( www.ipaq.ki.se). Finally, total cholesterol (< 200 mg/dL and ≥ 200 mg/dL) and type 2 diabetes, defined as fasting glucose ≥ 126 mg/dL or previous diagnosis made by a physician, were also included.

### Procedures

Recruitment of participants was conducted by community health workers utilizing standardized tools. Questionnaires were based on the World Health Organization (WHO) STEPwise approach to surveillance (STEPs), validated in a pilot study and previously published. ^20^ Fieldworker’s training included application of informed consent and questionnaires, and the attainment of clinical measurements using appropriate and calibrated methods. Blood pressure was measure in seated position after a resting period of five minutes. Measures were done by triplicate using an automated device (OMRON HEM-780) and the average of the second and third measurements was used to define hypertension. Laboratory assessments were performed on venous samples taken in the morning after a minimum of eight hours (maximum 12 hours) of fasting. Total cholesterol was measured in serum, and fasting glucose was measured in plasma using a Cobas ^®^ 6000 Modular Platform automated analyser and reagents supplied by Roche Diagnostics.

### Statistical analysis

STATA 16 for Windows (Stata Corp, College Station TX, US; RRID:SCR_012763) was used for statistical analysis. An open-access alternative that can provide an equivalent function is the R stats package (R Project for Statistical Computing, RRID:SCR_001905). Sociodemographic, lifestyle behavioural and anthropometric variables were described according to each definition of blood pressure levels (JNC-7 and ACC/AHA 2017) using the Chi-squared test. Variables were also described according to vital status using the Log-rank test. The Kaplan-Meier test was used to assess the bivariate association between variables of interest (i.e., JNC-7 and ACC/AHA 2017 definitions and 10-year mortality). The assumption of proportional hazards was assessed graphically and post-hoc analysis using the Schoenfeld residuals. Crude and adjusted Cox regression models were used to estimate the strength of the association between variables of interest, reporting hazard ratios (HR) and 95% confidence intervals (95% CI). Akaike and Bayesian information criteria (AIC and BIC) as well as the Nelson-Aalen graphs were utilized to compare both blood pressure level definitions and their impact on mortality.

### Ethics

The original PERU MIGRANT Study was approved by Institutional Review Boards (IRB) at Universidad Peruana Cayetano Heredia (approval codes: 51103, 60014 and 64094) in Peru and London School of Hygiene and Tropical Medicine (approval code: 5115) in UK. Follow-up was approved by the IRB at the UPCH only. Written informed consent was given by study participants prior to starting research activities. Permission was obtained to use personal identifiers to link participant’s information with vital status records; and only deidentified and anonymized data was used for publication. ^22^ The protocol for this secondary data analysis was approved by the ethics committee at Universidad Peruana de Ciencias Aplicadas (approval code: PI178-17) in Lima, Peru.

## Results

### Characteristics of the study population at baseline

A total of 989 participants were enrolled at baseline, but 13 (1.3%) were excluded as no mortality information was available at the end of the study. Thus, only 976 were included in further analyses. Of them, 196 (20.1%) were rural, 582 (59.6%) migrants, and 198 (20.3%) were urban dwellers, have a mean age of 60.4 (SD: 11.4), and 513 (52.6%) were women.

Hypertension prevalence at baseline almost doubled from 16.0% (95% CI 13.7%–18.4%) to 31.3% (95% CI 28.4%–34.3%) using the JNC-7 and ACC/AHA 2017 guidelines, respectively. Sex, age, population group, obesity, and type 2 diabetes mellitus were variables associated with blood pressure levels using both definitions ( Table 1 and Table 2).

**Table 1.** Characteristics of the study population by blood pressure levels according to the Seventh Report of the Joint National Committee on Prevention, Detection, Evaluation, and Treatment of High Blood Pressure (JNC-7).

	Blood pressure level			p-value *
	Normal	Pre-hypertension	Hypertension	
	(n = 508)	(n = 312)	(n = 156)	
Sex				
Men	181 (35.6%)	206 (66.0%)	76 (48.7%)	<0.001
Age				
30–49 years	352 (69.3%)	164 (52.6%)	43 (27.6%)	<0.001
50+ years	156 (30.7%)	148 (47.4%)	113 (72.4%)	
Education level				
<7 years	237 (46.7%)	148 (47.6%)	86 (55.1%)	0.18
7+ years	270 (53.3%)	163 (52.4%)	70 (44.9%)	
Socioeconomic status				
Low	226 (44.5%)	134 (43.0%)	64 (41.0%)	0.54
Middle	113 (22.2%)	79 (25.3%)	45 (28.9%)	
High	169 (33.3%)	99 (31.7%)	47 (30.1%)	
Population group				
Rural	105 (20.7%)	68 (21.8%)	23 (14.7%)	<0.001
Rural-to-urban migrant	316 (62.2%)	192 (61.5%)	74 (47.4%)	
Urban	87 (17.1%)	52 (16.7%)	59 (37.8%)	
Daily smoking				
Yes	14 (2.8%)	9 (2.9%)	10 (6.4%)	0.08
Alcohol use				
High consumption	38 (7.5%)	33 (10.6%)	15 (9.6%)	0.29
Physical activity				
Low levels	132 (26.2%)	72 (23.2%)	48 (31.2%)	0.18
Obesity				
BMI ≥ 30 kg/m ^2^	90 (17.7%)	57 (18.3%)	49 (31.4%)	0.001
Total cholesterol				
≥200 mg/dL	124 (24.4%)	117 (37.6%)	63 (40.4%)	<0.001
Type 2 diabetes				
Yes	11 (2.2%)	15 (4.8%)	13 (8.4%)	0.002

* Chi-squared test was used for comparisons.

**Table 2.** Characteristics of the study population by blood pressure levels according to the American College of Cardiology and the American Heart Association (ACC/AHA) 2017.

	Blood pressure level				p-value *
	Normal	Elevated	Stage 1 hypertension	Stage 2 hypertension	
	(n = 508)	(n = 163)	(n = 149)	(n = 156)	
Sex					
Men	181 (35.6%)	104 (63.8%)	102 (68.4%)	76 (48.7%)	<0.001
Age					
30–49 years	352 (69.3%)	80 (49.1%)	84 (56.4%)	43 (27.6%)	<0.001
50+ years	156 (30.7%)	83 (50.9%)	65 (43.6%)	113 (72.4%)	
Education level					
<7 years	237 (46.7%)	80 (49.4%)	68 (45.6%)	86 (55.1%)	0.27
7+ years	270 (53.3%)	82 (50.6%)	81 (54.4%)	70 (44.9%)	
Socioeconomic status					
Low	226 (44.5%)	73 (44.8%)	61 (41.0%)	64 (41.0%)	0.44
Middle	113 (22.2%)	35 (21.5%)	44 (29.5%)	45 (28.9%)	
High	169 (33.3%)	55 (33.7%)	44 (29.5%)	47 (30.1%)	
Population group					
Rural	105 (20.7%)	37 (22.7%)	31 (20.8%)	23 (14.7%)	<0.001
Rural-to-urban migrant	316 (62.2%)	104 (63.8%)	88 (59.1%)	74 (47.4%)	
Urban	87 (17.1%)	22 (13.5%)	30 (20.1%)	59 (37.8%)	
Daily smoking					
Yes	14 (2.8%)	3 (1.8%)	6 (4.1%)	10 (6.4%)	0.10
Alcohol use					
High consumption	38 (7.5%)	18 (11.0%)	15 (10.1%)	15 (9.6%)	0.47
Physical activity					
Low levels	132 (26.2%)	39 (24.2%)	33 (22.2%)	48 (31.2%)	0.31
Obesity					
BMI ≥ 30 kg/m ^2^	90 (17.7%)	31 (19.0%)	26 (17.5%)	49 (31.4%)	0.001
Total cholesterol					
≥200 mg/dL	124 (24.4%)	61 (37.7%)	56 (37.6%)	63 (40.4%)	<0.001
Type 2 diabetes					
Yes	11 (2.2%)	5 (3.1%)	10 (6.7%)	13 (8.4%)	0.002

* Chi-squared test was used for comparisons.

### Mortality and associated factors

A total of 63 (6.4%) participants died during the 10-year follow-up with 9992.6 person-years of follow-up and a mortality rate of 3.6 (95% CI 2.4–4.7) per 1000 person-years. In the bivariate model, men, older individuals, those with lower education, those with lower socioeconomic status, and having type 2 diabetes mellitus had an increased risk of 10-year mortality ( Table 3).

**Table 3.** Characteristics of the study population by vital status.

	Vital status		p-value *
	Alive (n = 913)	Dead (n = 63)	
Sex			
Women	491 (95.7%)	22 (4.3%)	0.004
Men	422 (91.1%)	41 (8.9%)	
Age			
30–49 years	551 (98.6%)	8 (1.4%)	<0.001
50+ years	362 (86.8%)	55 (13.2%)	
Education level			
<7 years	428 (90.9%)	43 (9.1%)	0.001
7+ years	483 (96.0%)	20 (4.0%)	
Socioeconomic status			
Low	386 (91.0%)	38 (9.0%)	0.01
Middle	229 (96.6%)	8 (3.4%)	
High	298 (94.6%)	17 (5.4%)	
Population group			
Rural	178 (90.8%)	18 (9.2%)	0.19
Rural-to-urban migrant	550 (94.5%)	32 (5.5%)	
Urban	185 (93.4%)	13 (6.6%)	
Daily smoking			
No	880 (93.5%)	61 (6.5%)	0.95
Yes	31 (93.9%)	2 (6.1%)	
Alcohol use			
Low consumption	834 (93.7%)	56 (6.3%)	0.52
High consumption	79 (91.9%)	7 (8.1%)	
Physical activity			
High/moderate levels	667 (93.2%)	49 (6.8%)	0.35
Low levels	239 (94.8%)	13 (5.2%)	
Obesity			
BMI < 30 kg/m ^2^	727 (93.2%)	53 (6.8%)	0.39
BMI ≥ 30 kg/m ^2^	186 (94.9%)	10 (5.1%)	
Total cholesterol			
<200 mg/dL	623 (92.9%)	48 (7.1%)	0.13
≥200 mg/dL	290 (95.4%)	14 (4.6%)	
Type 2 diabetes			
No	879 (93.9%)	57 (6.1%)	0.02
Yes	33 (84.6%)	6 (15.4%)	

* P-value estimated using Log-rank test.

### Blood pressure levels and 10-year mortality

There was evidence of an association between hypertension-related categories and all-cause mortality risk ( Table 4). Using the JNC-7 guideline, those with pre-hypertension and hypertension had 2.1-fold and 5.1-fold increased risk of death, respectively. On the other hand, using the ACC/AHA 2017 definition, stage-1 and stage-2 hypertension were associated with a 2.8- and 5.1-fold increase in the risk of mortality. There was no evidence of an association between the ACC/AHA 2017’s elevated blood pressure category and mortality.

**Table 4.** Association between blood pressure levels by Seventh Report of the Joint National Committee on Prevention, Detection, Evaluation, and Treatment of High Blood Pressure (JNC-7) and American College of Cardiology and the American Heart Association (ACC/AHA) 2017 definition and 10-year mortality: crude and adjusted Cox models.

Blood pressure definition	Vital status				
	Alive	Dead	Crude model	Adjusted model 1 *	Adjusted model 2 **
	(n = 913)	(n = 63)	HR (95% CI)	HR (95% CI)	HR (95% CI)
**JNC-7**					
Normal	497 (97.8%)	11 (2.2%)	1 (Reference)	1 (Reference)	1 (Reference)
Pre-hypertension	289 (92.6%)	23 (7.4%)	**3.50 (1.71–7.19)**	**2.19 (1.05–4.57)**	**2.14 (1.02–4.54)**
Hypertension	127 (81.4%)	29 (18.6%)	**9.4 (4.71–18.89)**	**5.08 (2.47–10.42)**	**5.08 (2.45–10.53)**
**ACC/AHA 2017**					
Normal	497 (97.8%)	11 (2.2%)	1 (Reference)	1 (Reference)	1 (Reference)
Elevated blood pressure	153 (93.9%)	10 (6.1%)	**2.87 (1.21–6.75)**	1.70 (0.72–4.06)	1.66 (0.68–4.07)
Stage 1 hypertension	136 (91.3%)	13 (8.7%)	**4.22 (1.89–9.42)**	**2.84 (1.25–6.48)**	**2.83 (1.23–6.53)**
Stage 2 hypertension	127 (81.4%)	29 (18.6%)	**9.44 (4.71–18.89)**	**5.08 (2.47–10.42)**	**5.09 (2.45–10.55)**

Bold estimates are significant at p < 0.05 level.

* Adjusted model 1 was controlled by age, sex, population group, education level, and socioeconomic status.

** Adjusted model 2 was controlled by age, sex, population group, education level, socioeconomic status, daily smoking, alcohol use, physical activity, obesity status, total cholesterol, and type 2 diabetes mellitus.

When comparing adjusted models using AIC and BIC, models were very similar (AIC was 755.1 for JNC-7 vs. 755.6 for ACC/AHA 2017, whereas BIC was 828.1 for JNC-7 vs. 833.5 for ACC/AHA 2017), highlighting no difference between models.

Should hypertension cases be treated and appropriately controlled, only 46.0% (29/63) deaths would have been avoided using the JNC-7 definition; however, this estimate would increase to 66.7% (42/63) using the ACC/AHA 2017 guidelines.

## Discussion

### Main findings

High blood pressure levels increased the risk of 10-year all-cause mortality, and our estimates showed similar long-term effect sizes across blood pressure categories using two different guidelines. As countries move into better universal health coverage, primary prevention and access to medications should be secured to reduce the health burden of raised blood pressure. However, how countries prepare and secure resources to successfully meet the challenges of hypertension will depend on how this is defined. There was a remarkable difference on hypertension prevalence depending on whether the JNC-7 or the ACC/AHA 2017 definition was followed, but the latter definition would avoid approximately 20% more deaths than the JNC-7 guideline. This carries relevant implications and repercussions for patients and health systems. Should the ACC/AHA 2017 definition be adopted because this will require securing treatment for a substantial larger population with the costs and challenges it entails.

### Comparison with previous studies

In the US, the SPRINT Study reported that the ACC/AHA 2017 definition significantly increased the prevalence of patients with hypertension and identified more patients who will experience adverse cardiovascular events. ^7^ However, it can be argued that information came from a clinical trial, which may have included more high-risk patients than in the general population; also, participants in the SPRINT Study were followed-up for 3.3 years. Conversely, we conducted a population-based 10-year follow-up study, advancing the evidence for the general population.

Because of data availability, we could not assess cardiovascular mortality; nonetheless, it is likely that we would have seen a similar – or even larger – effect as the one herein reported for all-cause mortality. In a pooled analysis of prospective cohorts conducted in China, ^15^ the ACC/AHA 2017 stage 1 hypertension was associated with an increased risk of cardiovascular disease mortality; notably, another cohort study, with 20 years of follow-up, did not find such association in rural dwellers in the same country. ^18^ The difference could be explained by different risk factor profiles in rural areas, or presumably lower levels of risk factors over twenty years ago. Using the National Health and Nutrition Examination Surveys between 2003 and 2014, a study found that the ACC/AHA 2017 guidelines would increase the proportion of stroke survivors in the US compared to the JNC-7 definition. ^23^ Thus, there is a potential benefit of applying the ACC/AHA 2017 guidelines, although this needs to be verified in different population groups.

### Public health relevance

The ACC/AHA 2017 guidelines radically proposed to change definitions of blood pressure levels, with pre-hypertension split into two categories: elevated blood pressure and stage I hypertension. Multiple authors have questioned this change, and pinpointed that hypertension prevalence would increase, pharmacotherapy of hypertension will start at a lower blood pressure level, and the threshold for hypertension control will decrease. ^10^^,^^24^ Thus, cases of stage I hypertension, previously classified as pre-hypertension in JNC-7, will start treatment with an initial anti-hypertensive drug if estimated 10-year cardiovascular risk is ≥ 10%, ^6^ but CV risk scores have showed poor concordance in Latin America populations ^25^; whereas those in stage II hypertension would start with two anti-hypertensive drugs. ^26^^–^^28^ In support of these concerns, a study showed that hypertension prevalence would increase by 40% in the US. ^10^ Similarly in Peru, using information from a population-based survey, the prevalence of hypertension would increase from 14% to 32%. ^12^ Peru is a middle-income country with a fragile and fragmented healthcare system, with poor response to the challenges of chronic conditions. Increasing the number of people with hypertension may benefit those with blood pressure levels in the range 130–139/80–89 mm Hg, but would represent a major investment so that these patients can receive adequate treatment. A thoroughly planned and balanced policy would be needed to provide care to those who most needed it. A combination of population-wide interventions, ^29^ along with high-risk stratification may be considered.

As the risk of coronary artery disease and stroke rise progressively increases as blood pressure increases above 115/75 mm Hg, ^30^ the beginning of antihypertensive therapy will certainly have advantages, especially the reduction of patient’s complications and mortality. ^31^ However, there will be an increase of primary care costs, which can be more deleterious in resource-constrained settings. A recent study conducted in the US has estimated that reaching the goals of the ACC/AHA 2017 guidelines will reduce 610,000 cardiovascular events and avoid 334,000 total deaths per year among adults 40 years and older. ^32^ Nevertheless, the potential increase of adverse events related to the use of anti-hypertensive drugs should be also considered ^33^ as well as a substantial number of hypertension cases giving up or taking medication irregularly. Thus, although the adoption of ACC/AHA 2017 guidelines may seem pertinent in term of complications and mortality reduction, Peru as well as other low- and middle-income countries, may not be prepared for this scenario.

### Strengths and limitations

This study takes advantage of an ongoing population-based cohort study conducted in a resource-constrained setting with three different population groups to evaluate the impact of two definitions of high blood pressure levels and 10-year mortality. However, this study has some limitations that should be highlighted. First, due to data availability, this study analysed all-cause mortality as outcome instead of assessing cardiovascular mortality. Since blood pressure increases the risk of cardiovascular events and mortality, we can speculate that the association of interest will be stronger and probably did not vary between hypertension definitions as in our analysis. Second, diet patterns and salt consumption, two potential confounders, were not included in our models as they were not available. Finally, we did not assess the potential effect of anti-hypertensive drugs on mortality due to limited sample size.

## Conclusions

Blood pressures levels under two different definitions increased the risk of 10-year all-cause mortality. Hypertension prevalence doubled using the ACC/AHA 2017 compared to the JNC-7 definition. The choice of blood pressure cut-offs to classify hypertension categories need to be balanced against the patient’s benefit and the capacities of the health system to adequately handle a large proportion of new patients. Cardiovascular disease prevention, and, in particular, the prevention of blood pressure-related mortality, will benefit from the estimates reported in this study to adequately inform local decision making, which in addition to disease burden should recognize balance benefits and risks within existing capacities to secure and guarantee adequate and effective treatment for all the new patients with raised blood pressure.

## Data availability

### Underlying data

Figshare: Underlying data for ‘Blood pressure and 10-year all-cause mortality: Findings from the PERU MIGRANT Study’, ‘PERU MIGRANT Study’, https://doi.org/10.6084/m9.figshare.16811350.v3. ^22^

This project includes the following underlying data:

- PERU MIGRANT Dataset (mortality).csv
- Dictionary.txt

Data are available under the terms of the Creative Commons Attribution 4.0 International license (CC-BY 4.0).

## Author contributions

Aida Hidalgo-Benites: conceptualization, data curation, formal analysis, investigation, methodology, writing original draft, and writing review and editing.

Valeria Senosain-Leon: conceptualization, data curation, formal analysis, investigation, methodology, writing original draft, and writing review and editing.

Rodrigo M. Carrillo-Larco: data curation, formal analysis, investigation, methodology, validation, and writing review and editing.

Andrea Ruiz-Alejos: data curation, formal analysis, funding acquisition, investigation, methodology, and writing review and editing.

Robert H. Gilman: funding acquisition, investigation, methodology, supervision, validation, and writing review and editing.

Liam Smeeth: funding acquisition, investigation, methodology, supervision, validation, and writing review and editing.

J. Jaime Miranda: conceptualization, funding acquisition, investigation, methodology, supervision, validation, and writing review and editing.

Antonio Bernabe-Ortiz: conceptualization, data curation, formal analysis, funding acquisition, investigation, methodology, supervision, and writing review and editing.
