# Supplementary material for: Blood pressure and 10-year all-cause mortality: Findings from the PERU MIGRANT Study
Source: F1000Res. 2023 Nov 20;10:1134. Originally published 2021 Nov 9. [Version 4] doi: 10.12688/f1000research.73900.4 (PMC10690031; doi:10.12688/f1000research.73900.4)
Supplement: Peer review history

# Reviewer report: Reviewer response for version 4

**DOI:** 10.5256/f1000research.158922.r224052

**Published:** 2023-11-30

**Authors:** Margarita Gutierrez

**Affiliations:** 1 University of the Philippines Manila College of Pharmacy, Manila, Philippines

No further comments.

Is the work clearly and accurately presented and does it cite the current literature?

Yes

If applicable, is the statistical analysis and its interpretation appropriate?

Yes

Are all the source data underlying the results available to ensure full reproducibility?

Yes

Is the study design appropriate and is the work technically sound?

Yes

Are the conclusions drawn adequately supported by the results?

Yes

Are sufficient details of methods and analysis provided to allow replication by others?

Yes

Reviewer Expertise:

Pharmacy, Social and Administraive pharmacy

I confirm that I have read this submission and believe that I have an appropriate level of expertise to confirm that it is of an acceptable scientific standard.

# Reviewer report: Reviewer response for version 4

**DOI:** 10.5256/f1000research.158922.r224053

**Published:** 2023-11-27

**Authors:** Annika Hoyer, Dina Voeltz

**Affiliations:** 1 Biostatistics and Medical Biometry, Medical School OWL, Bielefeld University, Bielefeld, Germany

The authors have responded and acted well to the points and comments raised to the previous version of the manuscript.

 Hence, the revised paper has been satisfactorily improved and modified and we have no further comments.

Is the work clearly and accurately presented and does it cite the current literature?

Partly

If applicable, is the statistical analysis and its interpretation appropriate?

Partly

Are all the source data underlying the results available to ensure full reproducibility?

Yes

Is the study design appropriate and is the work technically sound?

Yes

Are the conclusions drawn adequately supported by the results?

Partly

Are sufficient details of methods and analysis provided to allow replication by others?

Partly

Reviewer Expertise:

Biostatistics, Epidemiology, Diabetes, Chronic Diseases

We confirm that we have read this submission and believe that we have an appropriate level of expertise to confirm that it is of an acceptable scientific standard.

# Reviewer report: Reviewer response for version 3

**DOI:** 10.5256/f1000research.146604.r221681

**Published:** 2023-11-09

**Authors:** Margarita Gutierrez

**Affiliations:** 1 University of the Philippines Manila College of Pharmacy, Manila, Philippines

I encourage the author to address the following:

1. **Sample Size and Generalizability:** While the study provides valuable insights, it is important to ensure that the sample size and characteristics of the study population are representative of the broader population. A larger and more diverse sample could enhance the generalizability of the findings.
2. **Confounding Factors:** The study controlled for confounding factors, but it is essential to consider potential unmeasured confounders that might affect the relationship between blood pressure and mortality. A more comprehensive assessment of confounding variables, including lifestyle factors and comorbidities, would strengthen the study.
3. **Long-Term Follow-Up:** Ten years is a substantial follow-up period, but extending the observation period further could provide additional insights into the relationship between blood pressure and long-term mortality.
4. **Clinical Implications:** The authors should provide recommendations for healthcare practitioners based on their findings. This could include guidance on blood pressure management strategies, especially considering the increased prevalence of hypertension when using ACC/AHA 2017 guidelines.
5. **Discussion of Potential Biases:** The study should address potential biases, such as selection bias or measurement bias, and explain how these were minimized or managed.
6. **Public Health Impact:** The study mentions the capacity of healthcare systems to manage a larger number of hypertensive patients. A discussion of the potential public health impact of increased hypertension prevalence and its associated resource implications would be valuable.

Is the work clearly and accurately presented and does it cite the current literature?

Yes

If applicable, is the statistical analysis and its interpretation appropriate?

Yes

Are all the source data underlying the results available to ensure full reproducibility?

Yes

Is the study design appropriate and is the work technically sound?

Yes

Are the conclusions drawn adequately supported by the results?

Yes

Are sufficient details of methods and analysis provided to allow replication by others?

Yes

Reviewer Expertise:

Pharmacy, Social and Administraive pharmacy

I confirm that I have read this submission and believe that I have an appropriate level of expertise to confirm that it is of an acceptable scientific standard, however I have significant reservations, as outlined above.

# Response

**Published:** 2023-11-13

**Authors:** Antonio Bernabe-Ortiz

**Affiliations:** Universidad Peruana Cayetano Heredia, Peru

**Sample Size and Generalizability: While the study provides valuable insights, it is important to ensure that the sample size and characteristics of the study population are representative of the broader population. A larger and more diverse sample could enhance the generalizability of the findings.**

***Response:*** This is a secondary analysis of an existing dataset. The idea of the original study was to compare the cardiometabolic profile of three different populations (urban, rural-to-urban migrants, and rural dwellers). Even, we tried to take a random sample of participants for each population group, such selection was done in specific study sites using an age- and sex-stratification approach. Based on that, it is not possible to increase the sample size of the study.

**Confounding Factors: The study controlled for confounding factors, but it is essential to consider potential unmeasured confounders that might affect the relationship between blood pressure and mortality. A more comprehensive assessment of confounding variables, including lifestyle factors and comorbidities, would strengthen the study.**

***Response:*** We agree with the reviewer. Our adjusted model was built using epidemiological criteria and information available in the dataset. As a result, we did not include variables, important to control the model, but not present in the database. We have clarified that in the Statistical analysis section. In addition, we have added a comment about that in the Strengths and Limitations section:

*“* *Second, some variables, relevant to understand the relationship between hypertension and all-cause mortality, were not available (e.g., diet patterns, salt consumption, among others).”*

**Long-Term Follow-Up: Ten years is a substantial follow-up period, but extending the observation period further could provide additional insights into the relationship between blood pressure and long-term mortality.**

***Response:*** As pointed out by the reviewer, extending observation period would be ideal, but currently, that is not possible. Available information was used for this manuscript.

**Clinical Implications: The authors should provide recommendations for healthcare practitioners based on their findings. This could include guidance on blood pressure management strategies, especially considering the increased prevalence of hypertension when using ACC/AHA 2017 guidelines.**

**Response:** We would like to add a comment to provide recommendation for healthcare practitioners. However, this is an observational study looking at the association between two different guidelines for hypertension definition and all-cause mortality, and not a clinical trial to do decision making. Nevertheless, we have included a couple of sentences have been added:

*“From the clinical perspective, a tailored follow-up of those individuals with pre-hypertension may be required to decide appropriate treatment, even high-risk stratification may be considered. In addition to that, a combination of population-wide interventions may be also needed.”*

**Discussion of Potential Biases: The study should address potential biases, such as selection bias or measurement bias, and explain how these were minimized or managed.**

***Response:*** Thanks for the suggestion. We have added that in the Strengths and Limitations section: *“* *Fourth, selection bias can be an issue as studied population groups were selected in specific sites and may not be totally representative of the general population. In addition, measurement bias may be present. However, standard procedures were used to assess blood pressure levels.”*

**Public Health Impact: The study mentions the capacity of healthcare systems to manage a larger number of hypertensive patients. A discussion of the potential public health impact of increased hypertension prevalence and its associated resource implications would be valuable.**

 Response: We have discussed this point in our manuscript. We have extended some topics:

*“* *Peru is a middle-income country with a fragile and fragmented healthcare system, with poor response to the challenges of chronic conditions. Increasing the number of people with hypertension may benefit those with blood pressure levels in the range 130–139/80–89 mm Hg, but would represent a major investment (i.e., human resources, budget and time) so that these patients can receive adequate treatment, management, and follow-up.”*

# Reviewer report: Reviewer response for version 2

**DOI:** 10.5256/f1000research.142808.r160523

**Published:** 2023-01-24

**Authors:** Annika Hoyer, Dina Voeltz

**Affiliations:** 1 Biostatistics and Medical Biometry, Medical School OWL, Bielefeld University, Bielefeld, Germany

The authors have responded and acted well to the points and comments raised to the previous version of the manuscript. However, we still have some open questions and remarks.

 1. We do now understand the age cut-off at year 30 and thank the authors for clarification. Nonetheless, we are wondering how the new categorization is justified. With regards to blood pressure and mortality, we would think that particularly the age group 60+ might be at high risk and thus, more detailed analysis (i.e. splitting into further age group 60-69, 70-79, etc.) might be of interest. We kindly ask the authors for more details on the stratification choice. Overall though, we still recommend including age as a covariate measured on a continuous scale to improve the power of the analysis.

 2. Current guidelines used to report the results of observational studies such as STROBE clearly state that inferential measures should not be used for description of the study population. In line with that, we strongly suggest to delete all p-values from tables 1-3 and to simply describe the characteristics of the study participants. Further, and also as advanced by current guidelines, the use of p-values is not an appropriate criterion for selecting which confounders to adjust for in the analysis. Consequently, we advocate that any statement about potential associations based on single p-values should be avoided.

 3. We thank the authors for rewriting the Statistical analysis section where they refer to the Kaplan-Meier estimator. In the text it now reads that the evaluation of the proportional hazards assumption is based on visual inspection of a graph. For higher transparency and better understanding, we would suggest to include the graph in the article or as supporting material. 

 4. We are pleased to see that the authors followed our recommendation and changed the term “bivariate model”. Though, the term “bivariable model” is still somewhat misleading and rather uncommon to use. We would suggest to simply refer to an “association”.

 5. As suggested by the authors, we have reviewed the Public health relevance section for more information on how their study provides input e.g. for local decision making or public health implications. From our point of view, the article still lacks precise guidance in which way the estimates/results from the study presented in the article can be used for decision making with respect to benefits and risks for patients with newly diagnosed hypertension. Consequently, we highly recommend that the authors explain the implications of their study in more detail and that they provide concrete ideas of possible future activities.

 6. In our first review we noted that the authors were not consistent in their writing and with the vocabulary used in the tables, e.g. "stage 1" vs. "stage-1" vs. "stage I" (same applies for “stage 2”). In the reviewed article we still find inconsistencies, particularly comparing the Public health relevance section with the rest of the text.

Is the work clearly and accurately presented and does it cite the current literature?

Partly

If applicable, is the statistical analysis and its interpretation appropriate?

Partly

Are all the source data underlying the results available to ensure full reproducibility?

Yes

Is the study design appropriate and is the work technically sound?

Yes

Are the conclusions drawn adequately supported by the results?

Partly

Are sufficient details of methods and analysis provided to allow replication by others?

Partly

Reviewer Expertise:

Biostatistics, Epidemiology, Diabetes, Chronic Diseases

We confirm that we have read this submission and believe that we have an appropriate level of expertise to confirm that it is of an acceptable scientific standard, however we have significant reservations, as outlined above.

# Response

**Published:** 2023-04-04

**Authors:** Antonio Bernabe-Ortiz

**Affiliations:** Universidad Peruana Cayetano Heredia, Peru

**1. We do now understand the age cut-off at year 30 and thank the authors for clarification. Nonetheless, we are wondering how the new categorization is justified. With regards to blood pressure and mortality, we would think that particularly the age group 60+ might be at high risk and thus, more detailed analysis (i.e., splitting into further age group 60-69, 70-79, etc.) might be of interest. We kindly ask the authors for more details on the stratification choice. Overall, though, we still recommend including age as a covariate measured on a continuous scale to improve the power of the analysis.**

***Response:*** The categorization of the age variable is due to the fact that the sampling approach was stratified by age in that way (i.e., 30-39, 40-49, 50-59, and 60+). For instance, even we can adjust for that variable in different forms (i.e., using different cut-offs or in a continuous fashion), the best way would be to adjust for the form the variable was stratified. This was explained in the “Settings and participants” subheading in the Methods section. In addition, this was added in the Statistical analysis subheading:

 “ *Potential confounders were selected under epidemiological criteria. Since the selection of the participant’s sample was stratified by age (i.e., 30-39, 40-49, 50-59, 60+), such variable was included in that form in adjusted models.*”

**2. Current guidelines used to report the results of observational studies such as STROBE clearly state that inferential measures should not be used for description of the study population. In line with that, we strongly suggest to delete all p-values from tables 1-3 and to simply describe the characteristics of the study participants. Further, and also as advanced by current guidelines, the use of p-values is not an appropriate criterion for selecting which confounders to adjust for in the analysis. Consequently, we advocate that any statement about potential associations based on single p-values should be avoided.**

***Response:*** We have deleted p-values from tables 1 to 3 as suggested. We have not selected confounders based on p-values, but in an epidemiological approach (pre-specified model).

**3. We thank the authors for rewriting the Statistical analysis section where they refer to the Kaplan-Meier estimator. In the text it now reads that the evaluation of the proportional hazard assumption is based on visual inspection of a graph. For higher transparency and better understanding, we would suggest to include the graph in the article or as supporting material.**

***Response:*** We have included the figure in the manuscript as requested. We believe the figure does not add more information. Thus, we think the figure should be included in online material but F1000Research does not accept supplemental material. We leave the final decision to include the figure to the reviewer and the editor.

**4. We are pleased to see that the authors followed our recommendation and changed the term “bivariate model”. Though, the term “bivariable model” is still somewhat misleading and rather uncommon to use. We would suggest to simply refer to an “association”.**

***Response:*** We have deleted the word bivariable from the text of the manuscript.

**5. As suggested by the authors, we have reviewed the Public health relevance section for more information on how their study provides input e.g. for local decision making or public health implications. From our point of view, the article still lacks precise guidance in which way the estimates/results from the study presented in the article can be used for decision making with respect to benefits and risks for patients with newly diagnosed hypertension. Consequently, we highly recommend that the authors explain the implications of their study in more detail and that they provide concrete ideas of possible future activities.**

***Response:*** This is an observational study looking at the association between two different guidelines for hypertension definition and all-cause mortality, and not a clinical trial to do decision making. However, according to our results, we have added some lines:

*“Treatment must be guaranteed for hypertension (using JNC-7) and stage 2 hypertension (ACC/AHA 2017) to reduce mortality risk. If this effect can be seen in pre-hypertension (JNC-7) and stage 1 hypertension (ACC/AHA 2017) should be further assessed.”*

**6. In our first review we noted that the authors were not consistent in their writing and with the vocabulary used in the tables, e.g. "stage 1" vs. "stage-1" vs. "stage I" (same applies for “stage 2”). In the reviewed article we still find inconsistencies, particularly comparing the Public health relevance section with the rest of the text.**

***Response:*** We have reviewed through manuscript, tables and figures to keep consistency.

# Reviewer report: Reviewer response for version 1

**DOI:** 10.5256/f1000research.77589.r144138

**Published:** 2022-07-22

**Authors:** Annika Hoyer

**Affiliations:** 1 Biostatistics and Medical Biometry, Medical School OWL, Bielefeld University, Bielefeld, Germany

In the manuscript at hand, the authors report results from a study evaluating the association between blood pressure and all-cause mortality. They aim to investigate whether there are differences in the association using two different definitions of hypertension: The Seventh Report of the Joint National Committee on Prevention, Detection, Evaluation, and Treatment of High Blood Pressure (JNC-7) and the more recent guideline of the American College of Cardiology and the American Heart Association (ACC/AHA). The main difference between them is that the JNC-7 guideline consists of three categories whereas the ACC/AHA guideline comprises four stages, leading to a change in the cut-off points for defining hypertension and the corresponding disease prevalence. Based on data from the PERU MIGRANT study, the authors found that there is evidence for an association between the different definitions of hypertension and mortality while accounting for potential confounders. This finding is in line with the results from previous studies, but has the advantage that it is based on a large population-based cohort study. As a main conclusion the authors recommend that the choice of cut-off values for defining hypertension should be balanced with respect to the patient's benefit and the capacities of the health system because of a rising number of people with hypertension using the ACC/AHA guideline.

 The article is well written and spreads light on the association between blood pressure and mortality depending on the cut-off for defining high blood pressure in the Peruian population. However, we have some comments and suggestions that may improve the article:

1. In the introduction, it was written that "prevalence of hypertension was estimated to be 24.1% and 20.1% in men and women, respectively". It would be helpful to add the year that corresponds to these prevalence's.
2. In the introduction, we would suggest to briefly discuss the reasons that initially led to a reformulation of the definition of hypertension, i.e. what were the motives of the ACC/AHA to change the cut-off levels, and why did they introduce four instead of three categories?
3. In the "Settings and participants" section, it remains unclear what is meant by an "age and stratified sampling approach" which is used to select participants of the PERU MIGRANT study. This should be clarified.
4. What is the reason for splitting age into two categories (< 50 vs. >= 50)? With respect to the power of the analysis, it would be far better to include age as a covariate measured on a continuous scale.
  We were also wondering about the reason for the age cut-off at year 30? Is there a reason why no children, adolescents and any other people below 30 years are excluded?
5. We would strongly recommend to delete all p-values from tables 1-3 and to purely report on the characteristics of the population. This would be in line with current guidelines used to report the results of observational studies (e.g. STROBE).
6. We are a bit confused about the term "Kaplan-Meier test" and would suggest that the authors explain in more detail what specific kind of statistical test they used. If they are interested in the association between the exposure and outcome, it would be also possible to assess the estimated hazard ratios (HR) and their confidence intervals based on the proportional hazard regression approach.
7. Drawing conclusions about potential associations between variables based on univariate p-values (tables 1-3) alone should be viewed critically because the PERU MIGRANT study is a cohort study. That means that there are indeed confounding variables that are not adjusted for in tables 1-3. Again, we would recommend to delete all p-values from tables 1-3, to simply describe the characteristics of the study participants and to avoid statements about potential associations based on single p-values.
8. The term "bivariate model" should be avoided when only the association between two variables is meant because in a bivariate analysis, the outcome consists of two variables.
9. With respect to the Cox model, we would suggest to interpret the HR in terms of hazards and not risks. That means, a HR of 5.1 indicates a 5.1-fold increased hazard (and not a risk). It should also be added compared to which group the hazard is increased (or decreased).
10. We were wondering if the authors took account for possible left truncation which maybe occurred because the minimum age of entry is 30 years.
11. When discussing the AIC and BIC for the models, the authors should be more precise and state which models were compared and which result refers to which comparison.
12. In the section where the authors compare their findings with other studies, the dates of the studies mentioned should be added.
13. In the introduction and conclusion, the authors highlight that three different population groups were separately considered. Though, in the result section this is not further taken up. We would recommend that the authors explain why the distinction is yet so important and if the findings of their study differ with regards to the different groups.
14. From our point of view, the conclusion that 46% of deaths could be avoided using the JNC-7 definition, and 66.7% using the ACC/AHA 2017 guideline if hypertension is treated, seem a bit speculative. Could deaths be really prevented if people are classified as being hypertensive? We think such a conclusion cannot be drawn from a cohort study and the analysis done by the authors because it is not known if hypertension was the reason for all reported deaths. Only associations are seen. This should be discussed in more detail. We would also suggest to weaken this conclusion.
15. We would suggest that the authors explain the implications of their study in more detail. As a concluding remark, they have written that their estimates can be used to inform local decision making. We were wondering how this can be achieved based on the estimated HRs because they do not differ that much between the two definitions of hypertension. It would be helpful to describe in which way these estimates can be used for decision making with respect to benefits and risks for patients with newly diagnosed hypertension.

Minor comments:

1. The authors may want to add a decimal separator (e.g. 1,000 person years).
2. With regards to abbreviations: They should be introduced when first used (e.g. page 9 "CV risk scores"), and only introduced when needed (e.g. STEPs is never referred to again). First, the full name should be spelled and then the abbreviation should be added in brackets (e.g. Systolic Blood Pressure Intervention Trial Study (SPRINT)). Abbreviations in the abstract should be avoided (e.g. HR).
3. The authors should be consistent in their writing and with the vocabulary used in the tables. E.g. "stage 1" vs. "stage-1" vs. "stage I" (same applies for “stage 2”)
  and "Las Pampas de San Juan de Miraflores" vs. "San Juan de Miraflores".
4. Abstract: The data of the PERU MIGRANT Study was used, not the analysis, we think.
5. Page 3, Settings and Participants: The sentence “In Lima, a highly urbanized city, Las Pampas de San Juan de Miraflores was selected as the urban environment,” should be rephrased as follows “Las Pampas de San Juan de Miraflores, a highly urbanized city in Lima, Peru, was selected as the urban environment....”.
6. Page 4: There is a typo in the Procedures section. It should be read “measured” instead of “measure” in the following sentence: “Blood pressure was measured in seated position after a resting period of five minutes.”
7. Page 4: Another typo is found in the Ethics section. Instead of “in UK” the authors should write “in the UK” at the end of the first sentence of the mentioned paragraph.
8. Page 7: When discussing the main findings, we would suggest reformulating the following sentence on page 7 to “High blood pressure levels have been found to increase the risk of 10-year all-cause mortality *(please add a reference) *…”.

Is the work clearly and accurately presented and does it cite the current literature?

Partly

If applicable, is the statistical analysis and its interpretation appropriate?

Partly

Are all the source data underlying the results available to ensure full reproducibility?

Yes

Is the study design appropriate and is the work technically sound?

Yes

Are the conclusions drawn adequately supported by the results?

Partly

Are sufficient details of methods and analysis provided to allow replication by others?

Partly

Reviewer Expertise:

Biostatistics, Epidemiology, Diabetes, Chronic Diseases

I confirm that I have read this submission and believe that I have an appropriate level of expertise to confirm that it is of an acceptable scientific standard, however I have significant reservations, as outlined above.

# Response

**Published:** 2023-01-02

**Authors:** Antonio Bernabe-Ortiz

**Affiliations:** Universidad Peruana Cayetano Heredia, Peru

**REVIEWER 1 - ANNIKA HOYER**

 The article is well written and spreads light on the association between blood pressure and mortality depending on the cut-off for defining high blood pressure in the Peruvian population. However, we have some comments and suggestions that may improve the article:

**In the introduction, it was written that "prevalence of hypertension was estimated to be 24.1% and 20.1% in men and women, respectively". It would be helpful to add the year that corresponds to these prevalence's.**

***Response:*** We have rewritten the sentence to clarify this point. Now, it reads: “ *…and the prevalence of hypertension in 2015 was estimated to be 24.1% and 20.1% in men and women, respectively.*”

**In the introduction, we would suggest to briefly discuss the reasons that initially led to a reformulation of the definition of hypertension, i.e., what were the motives of the ACC/AHA to change the cut-off levels, and why did they introduce four instead of three categories?**

***Response:*** Although the focus of the paper was not to define the motivations of the ACC/AHA to change the cut-off levels, we have introduced some lines regarding this point. Now, it reads: “ *In 2017, as part of the ongoing review process of full guidelines commissioned in about 6-year cycles, the American College of Cardiology and the American Heart Association (ACC/AHA 2017) changed the proposed cut-off points used for defining hypertension, …*”

**In the "Settings and participants" section, it remains unclear what is meant by an "age and stratified sampling approach" which is used to select participants of the PERU MIGRANT study. This should be clarified.**

***Response:*** Thanks for noticing these typos. We have corrected this section to better clarify this point. Now, it reads: “ *Participants were randomly selected using an age- (30–39, 40–49, 50–59, and 60+) and sex- stratified sampling approach, utilizing the most up-to-date census in the study area.*”

**What is the reason for splitting age into two categories (< 50 vs. >= 50)? With respect to the power of the analysis, it would be far better to include age as a covariate measured on a continuous scale.**

***Response: *** Age is considered as confounder in our models. However, we have changed the categorization of age in four categories based on the stratification of the sample (30-39, 40-49, 50-69, and 60+ years). We have accordingly modified paper results. Overall, HR reduced after controlling for age in 4 categories, but are yet consistent.

**We were also wondering about the reason for the age cut-off at year 30? Is there a reason why no children, adolescents and any other people below 30 years are excluded?**

***Response:*** The original study at baseline recruited participants aged 30 years and over, and this was done because cardiovascular conditions are most common among subjects aged ≥ 30 years. This is a secondary analysis of that data and for instance no data of other age group was available.

**We would strongly recommend to delete all p-values from tables 1-3 and to purely report on the characteristics of the population. This would be in line with current guidelines used to report the results of observational studies (e.g., STROBE).**

***Response: *** We have decided to keep the p-values in Tables 1 to 3 as they are important to show association of variables with the exposures and outcome of interest, and for instance, the role of potential confounders. We ask the editor to decide about this topic if relevant.

**We are a bit confused about the term "Kaplan-Meier test" and would suggest that the authors explain in more detail what specific kind of statistical test they used. If they are interested in the association between the exposure and outcome, it would be also possible to assess the estimated hazard ratios (HR) and their confidence intervals based on the proportional hazard regression approach.**

***Response:*** We have rewritten this section to clarify this point. It now reads: “ *A plot of the Kaplan-Meier estimator was used to evaluate the assumption of proportional hazards graphically, whereas such assessment was done in post-hoc analysis using the Schoenfeld residuals.*”

**Drawing conclusions about potential associations between variables based on univariate p-values (tables 1-3) alone should be viewed critically because the PERU MIGRANT study is a cohort study. That means that there are indeed confounding variables that are not adjusted for in tables 1-3. Again, we would recommend to delete all p-values from tables 1-3, to simply describe the characteristics of the study participants and to avoid statements about potential associations based on single p-values.**

***Response: *** We only described the findings in our Results section. To avoid confusion, we have rewritten this section as follows: “ *In both definitions, high blood pressure levels were more frequent among males, older subjects, migrant and urban dwellers, as well as those with obesity and those with type 2 diabetes mellitus*”.

**The term "bivariate model" should be avoided when only the association between two variables is meant because in a bivariate analysis, the outcome consists of two variables.**

***Response:*** We have changed the term by using “bivariable model”.

**With respect to the Cox model, we would suggest to interpret the HR in terms of hazards and not risks. That means, a HR of 5.1 indicates a 5.1-fold increased hazard (and not a risk). It should also be added compared to which group the hazard is increased (or decreased).**

***Response:*** We have rewritten this section as suggested: “ *Using the JNC-7 guideline and compared to those with normal blood pressure, those with pre-hypertension and hypertension had 2-fold and 3.4-fold increased hazard of death, respectively. On the other hand, using the ACC/AHA 2017 definition and compared to those with normal blood pressure, stage 1 and stage 2 hypertension were associated with a 2.5- and 3.5-fold increase in the hazard of mortality.*”

**We were wondering if the authors took account for possible left truncation which maybe occurred because the minimum age of entry is 30 years.**

***Response:*** Although left truncation can be present as minimum age at study entry was 30 years, we believe the effect can be negligible as the prevalence of hypertension in Peruvian population aged <30 years is very low (<3%). However, we have added that in the limitations section: “ *Third, although left truncation can be present as subjects <30 years were excluded, the potential effect of such limitation may be negligible as the prevalence of hypertension is low in that age group.*”

**When discussing the AIC and BIC for the models, the authors should be more precise and state which models were compared and which result refers to which comparison.**

***Response:*** We have clarified this point in the Statistical analysis section: “ *Using full-adjusted models, Akaike and Bayesian information criteria (AIC and BIC) as well as the Nelson-Aalen graphs were utilized to compare both blood pressure level definitions and their impact on mortality.*” In addition, this has been also clarified in the Results section: “ *When comparing adjusted models, AIC and BIC were very similar (AIC was 741.3 for JNC-7 vs. 742.2 for ACC/AHA 2017, whereas BIC was 824.1 for JNC-7 vs. 829.9 for ACC/AHA 2017), highlighting no difference between models.*”

**In the section where the authors compare their findings with other studies, the dates of the studies mentioned should be added.**

***Response:*** We have modified this section accordingly. We have added dates of recruitment of the studies.

**In the introduction and conclusion, the authors highlight that three different population groups were separately considered. Though, in the result section this is not further taken up. We would recommend that the authors explain why the distinction is yet so important and if the findings of their study differ with regards to the different groups.**

***Response:*** We have not included population groups in the Introduction section. However, we have included such information in the Methods section as it is needed to describe the cohort used in the analysis, and in the Discussion section, especially in the Strength and Limitations section. We have dropped this section as it does not affect the interpretation of the results.

**From our point of view, the conclusion that 46% of deaths could be avoided using the JNC-7 definition, and 66.7% using the ACC/AHA 2017 guideline if hypertension is treated, seem a bit speculative. Could deaths be really prevented if people are classified as being hypertensive? We think such a conclusion cannot be drawn from a cohort study and the analysis done by the authors because it is not known if hypertension was the reason for all reported deaths. Only associations are seen. This should be discussed in more detail. We would also suggest to weaken this conclusion.**

***Response:*** This section highlighted by the reviewer is not a conclusion, it is only a finding described taking into account the limitations of the study (only observational). However, based on the comment, we have deleted this section.

**We would suggest that the authors explain the implications of their study in more detail. As a concluding remark, they have written that their estimates can be used to inform local decision making. We were wondering how this can be achieved based on the estimated HRs because they do not differ that much between the two definitions of hypertension. It would be helpful to describe in which way these estimates can be used for decision making with respect to benefits and risks for patients with newly diagnosed hypertension.**

***Response:*** We believe all these topics are discussed in the Public Health Relevance section: (1) the increase in the prevalence of hypertension, and the use of medications would start at a lower blood pressure level, with the subsequent increase in care costs at the health system which will be translated to the patient. So, countries like Peru are not prepared for that change, and for instance, it will require appropriate strategies to face them. In addition, we also discuss in this section the increase of side effects due to the greater use of anti-hypertensive medication.

**The authors may want to add a decimal separator (e.g. 1,000 person years).**

***Response:*** This will depend on the journal style and not necessarily on us. The paper has been adapted for review by editors, so we believe this may be not important at all.

**With regards to abbreviations: They should be introduced when first used (e.g. page 9 "CV risk scores"), and only introduced when needed (e.g. STEPs is never referred to again). First, the full name should be spelled and then the abbreviation should be added in brackets (e.g., Systolic Blood Pressure Intervention Trial Study (SPRINT)). Abbreviations in the abstract should be avoided (e.g., HR).**

***Response:*** The meaning of SPRINT Study is in the introduction of the paper. The use of abbreviations in the abstract depends on the journal style. We have nevertheless described the meaning of such abbreviations.

**The authors should be consistent in their writing and with the vocabulary used in the tables. E.g. "stage 1" vs. "stage-1" vs. "stage I" (same applies for “stage 2”)**

**and "Las Pampas de San Juan de Miraflores" vs.** **"San Juan de Miraflores".**

***Response:*** We have modified the manuscript to be consistent with our terms.

**Abstract: The data of the PERU MIGRANT Study was used, not the analysis, we think.**

***Response:*** We have modified this section.

**Page 3, Settings and Participants: The sentence “In Lima, a highly urbanized city, Las Pampas de San Juan de Miraflores was selected as the urban environment,” should be rephrased as follows “Las Pampas de San Juan de Miraflores, a highly urbanized city in Lima, Peru, was selected as the urban environment....”.**

***Response:*** We have modified this section as suggested.

**Page 4: There is a typo in the Procedures section. It should be read “measured” instead of “measure” in the following sentence: “Blood pressure was measured in seated position after a resting period of five minutes.”**

***Response:*** We have modified this section as suggested.

**Page 4: Another typo is found in the Ethics section. Instead of “in UK” the authors should write “in the UK” at the end of the first sentence of the mentioned paragraph.**

***Response:*** We have modified this section as suggested.

**Page 7: When discussing the main findings, we would suggest reformulating the following sentence on page 7 to “High blood pressure levels have been found to increase the risk of 10-year all-cause mortality (please add a reference) …”.**

***Response:*** This sentence is not part of a reference but instead the interpretation of our findings. We have clarified that by adding an introduction phase as follows: “ *According to our findings, high blood pressure levels increased the risk of 10-year all-cause mortality, …*”

# Response

**Published:** 2023-01-04

**Authors:** Antonio Bernabe-Ortiz

**Affiliations:** Universidad Peruana Cayetano Heredia, Peru

The article is well written and spreads light on the association between blood pressure and mortality depending on the cut-off for defining high blood pressure in the Peruvian population. However, we have some comments and suggestions that may improve the article:

**In the introduction, it was written that "prevalence of hypertension was estimated to be 24.1% and 20.1% in men and women, respectively". It would be helpful to add the year that corresponds to these prevalence's.**

***Response:*** We have rewritten the sentence to clarify this point. Now, it reads: “ *…and the prevalence of hypertension in 2015 was estimated to be 24.1% and 20.1% in men and women, respectively.*”

**In the introduction, we would suggest to briefly discuss the reasons that initially led to a reformulation of the definition of hypertension, i.e., what were the motives of the ACC/AHA to change the cut-off levels, and why did they introduce four instead of three categories?**

***Response:*** Although the focus of the paper was not to define the motivations of the ACC/AHA to change the cut-off levels, we have introduced some lines regarding this point. Now, it reads: “ *In 2017, as part of the ongoing review process of full guidelines commissioned in about 6-year cycles, the American College of Cardiology and the American Heart Association (ACC/AHA 2017) changed the proposed cut-off points used for defining hypertension, …*”

**In the "Settings and participants" section, it remains unclear what is meant by an "age and stratified sampling approach" which is used to select participants of the PERU MIGRANT study. This should be clarified.**

***Response:*** Thanks for notice these typos. We have corrected this section to better clarify this point. Now, it reads: “ *Participants were randomly selected using an age- (30–39, 40–49, 50–59, and 60+) and sex- stratified sampling approach, utilizing the most up-to-date census in the study area.*”

**What is the reason for splitting age into two categories (< 50 vs. >= 50)? With respect to the power of the analysis, it would be far better to include age as a covariate measured on a continuous scale.**

***Response: *** Age is considered as confounder in our models. However, we have changed the categorization of age in four categories based on the stratification of the sample (30-39, 40-49, 50-69, and 60+ years). We have accordingly modified paper results. Overall, HR reduced after controlling for age in 4 categories, but are yet consistent.

**We were also wondering about the reason for the age cut-off at year 30? Is there a reason why no children, adolescents and any other people below 30 years are excluded?**

***Response:*** The original study at baseline recruited participants aged 30 years and over, and this was done because cardiovascular conditions are most common among subjects aged ≥ 30 years. This is a secondary analysis of that data and for instance no data of other age group was available.

**We would strongly recommend to delete all p-values from tables 1-3 and to purely report on the characteristics of the population. This would be in line with current guidelines used to report the results of observational studies (e.g., STROBE).**

***Response: *** We have decided to keep the p-values in Tables 1 to 3 as they are important to show association of variables with the exposures and outcome of interest, and for instance, the role of potential confounders. We ask the editor to decide about this topic if relevant.

**We are a bit confused about the term "Kaplan-Meier test" and would suggest that the authors explain in more detail what specific kind of statistical test they used. If they are interested in the association between the exposure and outcome, it would be also possible to assess the estimated hazard ratios (HR) and their confidence intervals based on the proportional hazard regression approach.**

***Response:*** We have rewritten this section to clarify this point. It now reads: “ *A plot of the Kaplan-Meier estimator was used to evaluate the assumption of proportional hazards graphically, whereas such assessment was done in post-hoc analysis using the Schoenfeld residuals.*”

**Drawing conclusions about potential associations between variables based on univariate p-values (tables 1-3) alone should be viewed critically because the PERU MIGRANT study is a cohort study. That means that there are indeed confounding variables that are not adjusted for in tables 1-3. Again, we would recommend to delete all p-values from tables 1-3, to simply describe the characteristics of the study participants and to avoid statements about potential associations based on single p-values.**

***Response: *** We only described the findings in our Results section. To avoid confusion, we have rewritten this section as follows: “ *In both definitions, high blood pressure levels were more frequent among males, older subjects, migrant and urban dwellers, as well as those with obesity and those with type 2 diabetes mellitus*”.

**The term "bivariate model" should be avoided when only the association between two variables is meant because in a bivariate analysis, the outcome consists of two variables.**

***Response:*** We have changed the term by using “bivariable model”.

**With respect to the Cox model, we would suggest to interpret the HR in terms of hazards and not risks. That means, a HR of 5.1 indicates a 5.1-fold increased hazard (and not a risk). It should also be added compared to which group the hazard is increased (or decreased).**

***Response:*** We have rewritten this section as suggested: “ *Using the JNC-7 guideline and compared to those with normal blood pressure, those with pre-hypertension and hypertension had 2-fold and 3.4-fold increased hazard of death, respectively. On the other hand, using the ACC/AHA 2017 definition and compared to those with normal blood pressure, stage 1 and stage 2 hypertension were associated with a 2.5- and 3.5-fold increase in the hazard of mortality.*”

**We were wondering if the authors took account for possible left truncation which maybe occurred because the minimum age of entry is 30 years.**

***Response:*** Although left truncation can be present as minimum age at study entry was 30 years, we believe the effect can be negligible as the prevalence of hypertension in Peruvian population aged <30 years is very low (<3%). However, we have added that in the limitations section: “ *Third, although left truncation can be present as subjects <30 years were excluded, the potential effect of such limitation may be negligible as the prevalence of hypertension is low in that age group.*”

**When discussing the AIC and BIC for the models, the authors should be more precise and state which models were compared and which result refers to which comparison.**

***Response:*** We have clarified this point in the Statistical analysis section: “ *Using full-adjusted models, Akaike and Bayesian information criteria (AIC and BIC) as well as the Nelson-Aalen graphs were utilized to compare both blood pressure level definitions and their impact on mortality.*” In addition, this has been also clarified in the Results section: “ *When comparing adjusted models, AIC and BIC were very similar (AIC was 741.3 for JNC-7 vs. 742.2 for ACC/AHA 2017, whereas BIC was 824.1 for JNC-7 vs. 829.9 for ACC/AHA 2017), highlighting no difference between models.*”

**In the section where the authors compare their findings with other studies, the dates of the studies mentioned should be added.**

***Response:*** We have modified this section accordingly. We have added dates of recruitment of the studies.

**In the introduction and conclusion, the authors highlight that three different population groups were separately considered. Though, in the result section this is not further taken up. We would recommend that the authors explain why the distinction is yet so important and if the findings of their study differ with regards to the different groups.**

***Response:*** We have not included population groups in the Introduction section. However, we have included such information in the Methods section as it is needed to describe the cohort used in the analysis, and in the Discussion section, especially in the Strength and Limitations section. We have dropped this section as it does not affect the interpretation of the results.

**From our point of view, the conclusion that 46% of deaths could be avoided using the JNC-7 definition, and 66.7% using the ACC/AHA 2017 guideline if hypertension is treated, seem a bit speculative. Could deaths be really prevented if people are classified as being hypertensive? We think such a conclusion cannot be drawn from a cohort study and the analysis done by the authors because it is not known if hypertension was the reason for all reported deaths. Only associations are seen. This should be discussed in more detail. We would also suggest to weaken this conclusion.**

***Response:*** This section highlighted by the reviewer is not a conclusion, it is only a finding described taking into account the limitations of the study (only observational). However, based on the comment, we have deleted this section.

**We would suggest that the authors explain the implications of their study in more detail. As a concluding remark, they have written that their estimates can be used to inform local decision making. We were wondering how this can be achieved based on the estimated HRs because they do not differ that much between the two definitions of hypertension. It would be helpful to describe in which way these estimates can be used for decision making with respect to benefits and risks for patients with newly diagnosed hypertension.**

***Response:*** We believe all these topics are discussed in the Public Health Relevance section: (1) the increase in the prevalence of hypertension, and the use of medications would start at a lower blood pressure level, with the subsequent increase in care costs at the health system which will be translated to the patient. So, countries like Peru are not prepared for that change, and for instance, it will require appropriate strategies to face them. In addition, we also discuss in this section the increase of side effects due to the greater use of anti-hypertensive medication.

**The authors may want to add a decimal separator (e.g. 1,000 person years).**

***Response:*** This will depend on the journal style and not necessarily on us. The paper has been adapted for review by editors, so we believe this may be not important at all.

**With regards to abbreviations: They should be introduced when first used (e.g. page 9 "CV risk scores"), and only introduced when needed (e.g. STEPs is never referred to again). First, the full name should be spelled and then the abbreviation should be added in brackets (e.g., Systolic Blood Pressure Intervention Trial Study (SPRINT)). Abbreviations in the abstract should be avoided (e.g., HR).**

***Response:*** The meaning of SPRINT Study is in the introduction of the paper. The use of abbreviations in the abstract depends on the journal style. We have nevertheless described the meaning of such abbreviations.

**The authors should be consistent in their writing and with the vocabulary used in the tables. E.g. "stage 1" vs. "stage-1" vs. "stage I" (same applies for “stage 2”)**

**and "Las Pampas de San Juan de Miraflores" vs.** **"San Juan de Miraflores".**

***Response:*** We have modified the manuscript to be consistent with our terms.

**Abstract: The data of the PERU MIGRANT Study was used, not the analysis, we think.**

***Response:*** We have modified this section.

**Page 3, Settings and Participants: The sentence “In Lima, a highly urbanized city, Las Pampas de San Juan de Miraflores was selected as the urban environment,” should be rephrased as follows “Las Pampas de San Juan de Miraflores, a highly urbanized city in Lima, Peru, was selected as the urban environment....”.**

***Response:*** We have modified this section as suggested.

**Page 4: There is a typo in the Procedures section. It should be read “measured” instead of “measure” in the following sentence: “Blood pressure was measured in seated position after a resting period of five minutes.”**

***Response:*** We have modified this section as suggested.

**Page 4: Another typo is found in the Ethics section. Instead of “in UK” the authors should write “in the UK” at the end of the first sentence of the mentioned paragraph.**

***Response:*** We have modified this section as suggested.

**Page 7: When discussing the main findings, we would suggest reformulating the following sentence on page 7 to “High blood pressure levels have been found to increase the risk of 10-year all-cause mortality (please add a reference) …”.**

***Response:*** This sentence is not part of a reference but instead the interpretation of our findings. We have clarified that by adding an introduction phase as follows: “ *According to our findings, high blood pressure levels increased the risk of 10-year all-cause mortality, …*”
